# The development of a clinical prediction model for response to methotrexate, tofacitinib, and etanercept in patients with Psoriatic Arthritis

**DOI:** 10.1186/s13075-025-03660-2

**Published:** 2025-10-27

**Authors:** F. T. Perton, M. L. M. Bentvelzen, S. Fadaei, J. N. Pouw, J. Spierings, H. E. Vonkeman, S. C. Mooij, L. G. Schipper, A. Herman, TOFA-PREDICT author group, Kavish J. Bhansing, Radjesh J. Bisoendial, Sandra T.A. van Bijnen, Lenny van Bon, Antoaneta C. Comarniceanu, Amin Herman, Z. Nazira Jahangier, Tim L.T.A. Jansen, Jacqueline S.L Kloth, Marc R. Kok, Arno W. R. van Kuijk, Emmerik F.A. Leijten, Shasti C. Mooij, Lydia G. Schipper, Astrid M. van Tubergen, Harald E. Vonkeman, Simone A. Vreugdenhil, Siska Wijngaarden, S. C. Mastbergen, P. M. J. Welsing

**Affiliations:** 1 Department of Rheumatology & Clinical Immunology, University Medical Center Utrecht, Utrecht University, Heidelberglaan 1003508 GA, Postbus 85090, Utrecht, The Netherlands; 2 Department of Rheumatology, Medisch Spectrum Twente, Enschede, The Netherlands; 3 Department of Psychology, Health and Technology, University of Twente, Enschede, The Netherlands; 4 Department of Rheumatology, Elisabeth-TweeSteden Hospital, Tilburg, The Netherlands; 5 Department of Rheumatology, St. Antonius Hospital, Utrecht, The Netherlands

**Keywords:** Psoriatic arthritis, DMARDs, JAK inhibitors, Interventional studies, Clinical trials and methods

## Abstract

**Background:**

The aim of this study was to use clinical data to develop a prediction model for treatment response, comparing tofacitinib to methotrexate or etanercept in individual patients with Psoriatic Arthritis.

**Methods:**

Data from the development cohort (*n* = 80) of the TOFA-PREDICT trial were used. The cohort included PsA patients naïve to disease-modifying antirheumatic drugs (DMARDs) randomised to tofacitinib (*n* = 20) or methotrexate (*n* = 20), and patients failing conventional synthetic DMARD (csDMARD) treatment randomised to add-on tofacitinib (*n* = 20) or etanercept (*n* = 20). Treatment response was defined as achievement of minimal disease activity at week 16. Elastic net regression was used to select relevant baseline predictors in the complete cohort and in treatment subgroups. Using Ridge regression with different modelling strategies, three prediction models were developed and compared. Based on performance, a final model was selected.

**Results:**

Increased Health Assessment Questionnaire score, increased tender joint count, increased Leeds Enthesitis Index score, decreased VAS global assessment by physician and previous Tumour Necrosis Factor alpha inhibitor treatment were selected as predictors for non-response. The final cross-validated model had an AUC-ROC of 0.76 and predicted clinically relevant differences in response to the compared treatments. The predicted probability of response was higher for methotrexate compared to tofacitinib in 85% of DMARD naïve patients. The predicted probability of response was higher for etanercept compared to tofacitinib in all patients failing csDMARD treatment.

**Conclusion:**

Our results support the use of baseline clinical data for prediction of response to different treatments. We intend to validate this prediction model and to assess the additional predictive value of imaging and multi-omics biomarkers in future analyses.

**Trial registration:**

EudraCT Trial registration number 2017–003900-28, registration date January 25th 2018.

**Supplementary Information:**

The online version contains supplementary material available at 10.1186/s13075-025-03660-2.

## Background

In patients with Psoriatic Arthritis (PsA), early initiation of effective disease-modifying antirheumatic drug (DMARD) treatment is important to alleviate symptoms, and to avoid irreversible joint damage and disability. [1, 2] In addition, DMARDS are reported to mitigate the risk of associated comorbidities, such as cardiovascular disease. [3].

Two factors complicate the selection of effective treatment in patients with PsA: the heterogeneous nature of PsA and the individual differences in response to treatment. [4, 5] For example, methotrexate is recommended as a first line of treatment for patients with PsA with predominantly peripheral arthritis, but reported response rates in treatment-naïve patients are relatively low, ranging from 22 to 43%. [6].

In the recommendations presented by the Group for Research and Assessment of Psoriasis and Psoriatic Arthritis (GRAPPA), the European alliance of associations for rheumatology (EULAR) and the American College of Rheumatology (ACR), treatment is guided by the involvement of clinical domains, and by the failing of previous treatment. [4, 7, 8] However, due to relatively poor response rates, this can lead to a trial-and-error approach and subsequently a delay in achieving remission. The individualized prediction and comparison of response to different DMARDS could help to identify the treatment with the highest probability of response. This could support treatment decisions for healthcare professionals and patients, expedite the achievement of remission, and ultimately improve the quality of life for the patient.

In the TOFA-PREDICT study, the final aim is to identify pretreatment profiles to predict response to tofacitinib, methotrexate and etanercept. As a first step, in the current analysis we use baseline clinical data to develop a prediction model to compare predicted responses to the different treatments for individual patients. Clinical data are easy and relatively cheap to acquire. A multitude of additional biomarkers (e.g. derived from cell phenotyping, transcriptomics, proteomics, metabolomics, imaging) may be considered, but for the sake of cost-effectiveness, the performance of complex multi-omics predictor variables should be assessed for added predictive value over clinical variables. A prediction model with clinical data can therefore serve as a benchmark for more complex models, which will be developed and validated at a later stage with data from the TOFA-PREDICT trial. [9].

In the past, several clinical variables have been reported to associate with treatment response in PsA. For Tumour Necrosis Factor alpha inhibitors (TNFi), many different clinical variables (Health Assessment Questionnaire (HAQ), C-reactive Protein (CRP), Body Mass Index (BMI), Disease Activity Index in Psoriatic Arthritis (DAPSA), gender, age, disease duration, etc.) have been reported as a predictor of response. [10–17] Sex, biological DMARD (bDMARD) history, and CRP were reported as predictors of response to Janus-Kinase inhibitor (JAKi) treatment. [18] For response to methotrexate, the presence of dactylitis and lower back pain are reported as predictors. [19] However, to our knowledge, a robust prediction model combining such clinical data to compare individualized treatment responses in PsA, has not previously been reported.

To summarize, our aim is to use baseline clinical data from the TOFA-PREDICT trial to develop a prediction model for individualized response to treatment with tofacitinib versus methotrexate in patients naïve to DMARD treatment, and add-on tofacitinib versus add-on etanercept in patients currently treated with a csDMARD. Such a model uses patient characteristics to predict which of two compared treatments has the highest probability of response.

## Methods

### Patients and treatment

The TOFA-PREDICT is an ongoing investigator initiated, phase III, multi-centre, open-label, randomised controlled trial, that started accrual of data in April 2018. In total 160 patients with PsA will be included, with at least two tender joints and at least two swollen joints at baseline. The study population consists of two groups: csDMARD naïve (DN) patients, and patients that previously had insufficient response to a csDMARD (csDMARD failure; DF) patients. DN patients are not currently being treated with csDMARD and have no history of csDMARD use. These patients are randomised (1:1) to receive methotrexate or tofacitinib. DF patients have active PsA despite current csDMARD use and are randomised (1:1) to receive add-on etanercept or tofacitinib. See also Supplementary Figure S1 for an overview of the study design. The TOFA-PREDICT trial protocol, including details on eligibility criteria, sample size, patient and public involvement, ethical approval, informed consent, and registration of the study can be found elsewhere. [9].

To enable validation, the TOFA-PREDICT study population (*n* = 160) was split into a development cohort (*n* = 80) and a validation cohort (*n* = 80). Clinical data of the development cohort were used for the current analysis. It consists of the first 20 patients for each of the four treatments. The patients were included in seven medical centres in the Netherlands: University Medical Center Utrecht, Medisch Spectrum Twente, Elisabeth TweeSteden Hospital, Maastricht University Medical Center, VieCuri Medical Center, Reade Medical Center and Gelre Hospital. Patients were also referred to the UMCU from other Dutch hospitals: St. Antonius Hospital, Maasstad Hospital, Gelderse Vallei Hospital, Diakonessenhuis, Tergooi Medical Center, Sint Maartenskliniek, St. Jansdal hospital and Ziekenhuisgroep Twente. The end of follow up for the development cohort was February 2023. The quality of the clinical data was monitored during accrual by Julius Clinical, a science-driven contract research organisation.

### Outcome measures

We used Minimal Disease Activity (MDA) after 16 weeks of treatment to define treatment response. [20] MDA is a validated outcome measure for PsA, that reflects its heterogeneous nature. Regarding the MDA criteria, the 66 and 68 joint counts were used for scoring swollen and tender joints, respectively. To assess psoriasis, we used body surface area (BSA). The Leeds Enthesitis Index (LEI) was used as a practical representation of enthesitis. [21] The researchers that assessed MDA were blinded for treatment allocation.

### Predictor variables

Clinical data were gathered by a research nurse and/or physician during the baseline visit, before initiation of study medication. Patient reported data was collected using online questionnaires. All assessors of the predictor variables were blinded to the outcome at week 16. All clinical predictor variables that were considered relevant are listed in Table 1. For predictor variables with a skewed distribution, in which associations with the outcome may more likely be non-linear, natural log or square root transformations were also evaluated for their association with response, and the best fitting predictor variable was used in subsequent analyses.

**Table 1:**
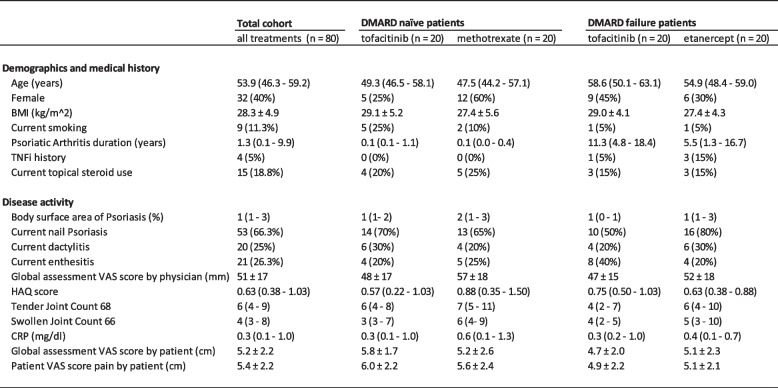
Baseline characteristics

Data is shown as n (%) for categorical variables, mean ± SD for normally distributed continuous variables, or median (IQR) for non-normally distributed continuous variables. For the variable current enthesitis all locations of the Leeds Enthesitis Score were assessed at baseline. DMARD: disease modifying antirheumatic drug; BMI: body mass index; TNFi: tumour necrosis factor alpha inhibitor; VAS: visual analog scale; HAQ: health assessment questionnaire; CRP: C-reactive protein.

### Statistical analysis

**Fig. 1:**
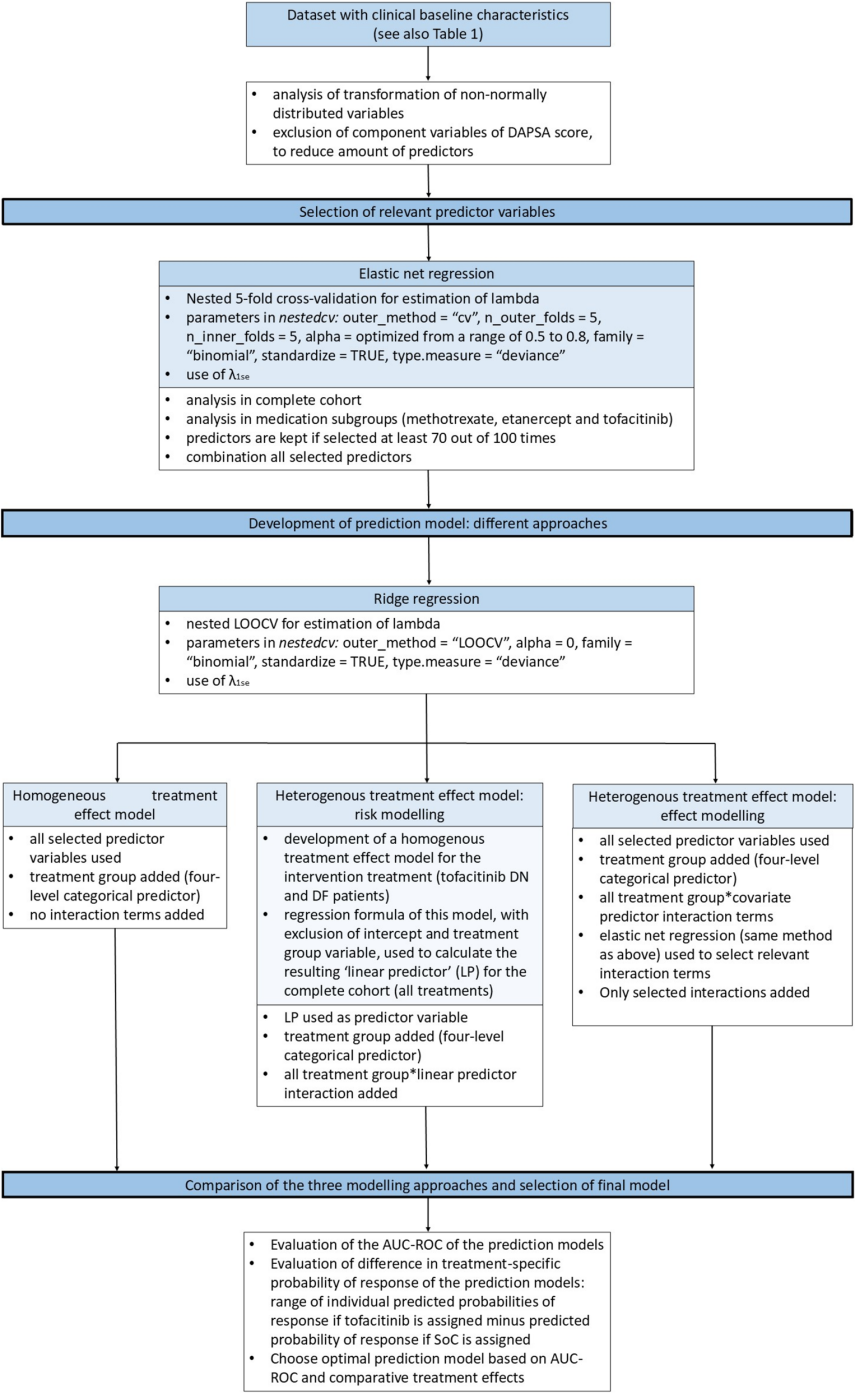
Overview of statistical analyses. Legend: DAPSA score: disease activity of psoriatic arthritis score; LOOCV: leave one out cross-validation; λ1se: the largest value for λ within one standard error of the λ with a minimum mean cross-validated error; DN: disease modifying antirheumatic drug naïve; DF: disease modifying antirheumatic drug failure; LP: linear predictor

For more information regarding the sample size calculation, we refer to the open protocol publication. [9] Fig. 1 gives an overview of the statistical analysis strategy for the development of the prediction model.

To select relevant predictor variables, we used elastic net regression. Relevant predictor variables were selected in the full cohort (*n* = 80), as well as in the subgroups of patients receiving; tofacitinib (*n* = 40), methotrexate (*n* = 20) or etanercept (*n* = 20). Predictor variables selected in any of these analyses were deemed relevant. Ridge regression was then performed to obtain a multivariate prediction model.

In penalised regression methods such as elastic net and Ridge, *p*-values are not routinely provided. Instead, cross-validation techniques were used to select relevant predictors. See supplementary information S2 for more details regarding elastic net, Ridge regression, and the used cross-validation methods.

Our main goal with individualised prediction of treatment response was to compare different treatment options and to select treatment with the highest probability of response for individual patients. To enable this, treatment group (as an indicator of the four treatment groups in the trial) was included as a four-level categorical covariate in the prediction model. Subsequently, three different approaches for the prediction of individualised treatment response were evaluated. [22].

Firstly, a homogeneous treatment effect model was developed. This is a simple model including all selected predictor variables and treatment group. This model assumes a constant (homogeneous) effect of treatment on the log-odds scale. However, since the predicted absolute difference in probability of response for tofacitinib and the compared treatment can still vary meaningfully between patients as a function of the predictor variables, this model may still be useful to personalise treatment.

The second approach, referred to as “risk modelling”, assumes a heterogeneous or differential treatment effect. [22] The basis of this approach was a general prediction model for response to tofacitinib developed by using all selected predictors in only the patients treated with tofacitinib (DN and DF). Subsequently a so called ‘linear predictor’ (LP) was derived for all patients (including patients that received methotrexate or etanercept), by multiplying the regression coefficients of this model with the corresponding predictor values, and excluding the intercept and treatment from this LP calculation. Subsequently, treatment as added categorical covariates, and the interaction between the LP and treatment were added to the model.

An assumption with risk modelling is that the predictive effect of the different predictor variables (represented by the LP) are modified by treatment in a similar way. However, taking this assumption can also be regarded a helpful data reduction method and is regarded a suitable and efficient way for small sample-sizes, as it may harness against problematic overfitting. This approach emphasises a comparison between the “intervention group” (tofacitinib treatment) versus “control group” (methotrexate or etanercept treatment). More details and the rationale behind this approach are described in supplementary information S3.

The third approach, ‘effect modelling’, also assumes a heterogeneous treatment effect. [22] In this strategy, all selected predictor variables, treatment group, and all treatment*predictor interaction terms are included in the model. In contrast to risk modelling, effect modelling does not assume similar modification of predictive effects of variables by treatment. However, it leads to a very large amount of regression coefficients to be estimated in limited data. Therefore, elastic net regression (similar approach as previously described) was used to select relevant interaction terms to incorporate in the effect model.

To compare the three different prediction models we assessed their performance and impact. Firstly, the area under the curve of the receiver operating characteristics (AUC-ROC) was assessed. Furthermore, we used the prediction models to calculate for each patient the probability of response for tofacitinib and for the compared treatment (methotrexate or etanercept). The differences between predicted probabilities of response for the compared treatments in individual patients were described for all the models. Since a larger difference in predicted response indicates a stronger preference for a specific treatment, the variation in these differences was used to compare the potential clinical impact of the different models.

The TRIPOD + AI checklist was used to report this study. [23] Analyses were performed using SPSS version 27.0 for Windows (IBM Corporation, Armonk, NY, USA) and R version 4.2.2 (2022–10-31 ucrt) for Windows. [24].

## Results

### Patient characteristics and predictor variables

In general, there were no major differences between the two randomised treatment groups for DMARD naïve (DN) patients, and for patients with insufficient response to csDMARD treatment (DF patients). Nail psoriasis, Tender Joint Count (TJC) 68, disease duration and prior TNFi use in the DF group, and sex in the DN group were somewhat different between the treatment arms (Table 1). The patient characteristics were representative of an early (DN) and established (DF) outpatient PsA population. There were no missing data for the predictor variables.

MDA was achieved in 50% of all patients. In DN patients, MDA was achieved in 50% of the patients receiving methotrexate and in 50% of the patients receiving tofacitinib. In DF patients, MDA was achieved in 60% of the patients receiving etanercept and in 40% of the patients receiving tofacitinib. Physical examination data at week 16 were missing for two DN patients randomised to tofacitinib, due to COVID-19 regulations. Data from the first subsequent visit (8 and 24 weeks later, respectively) were therefore combined with the available week 16 patient-reported outcomes (HAQ, VAS score for pain, VAS score for global activity) to calculate MDA at week 16.

### Selection of predictor variables

For BSA as a predictor a square root transformation was used, as this resulted in the best association with response. Other predictor variables were not transformed. The following variables were selected in the elastic net analyses: HAQ, LEI, TJC68, VAS global assessment by physician, and TNFi history. An increased HAQ score (indicating increased disability) was a relevant predictor for not achieving MDA in the complete cohort, in the tofacitinib subgroup and in the etanercept subgroup. Increased Leeds Enthesitis Index score, increased TJC68 and decreased VAS global assessment by physician were relevant predictors only in the methotrexate subgroup. Previous use of a TNFi was a relevant predictor only in the etanercept subgroup.

### Homogeneous treatment effect model

**Fig. 2:**
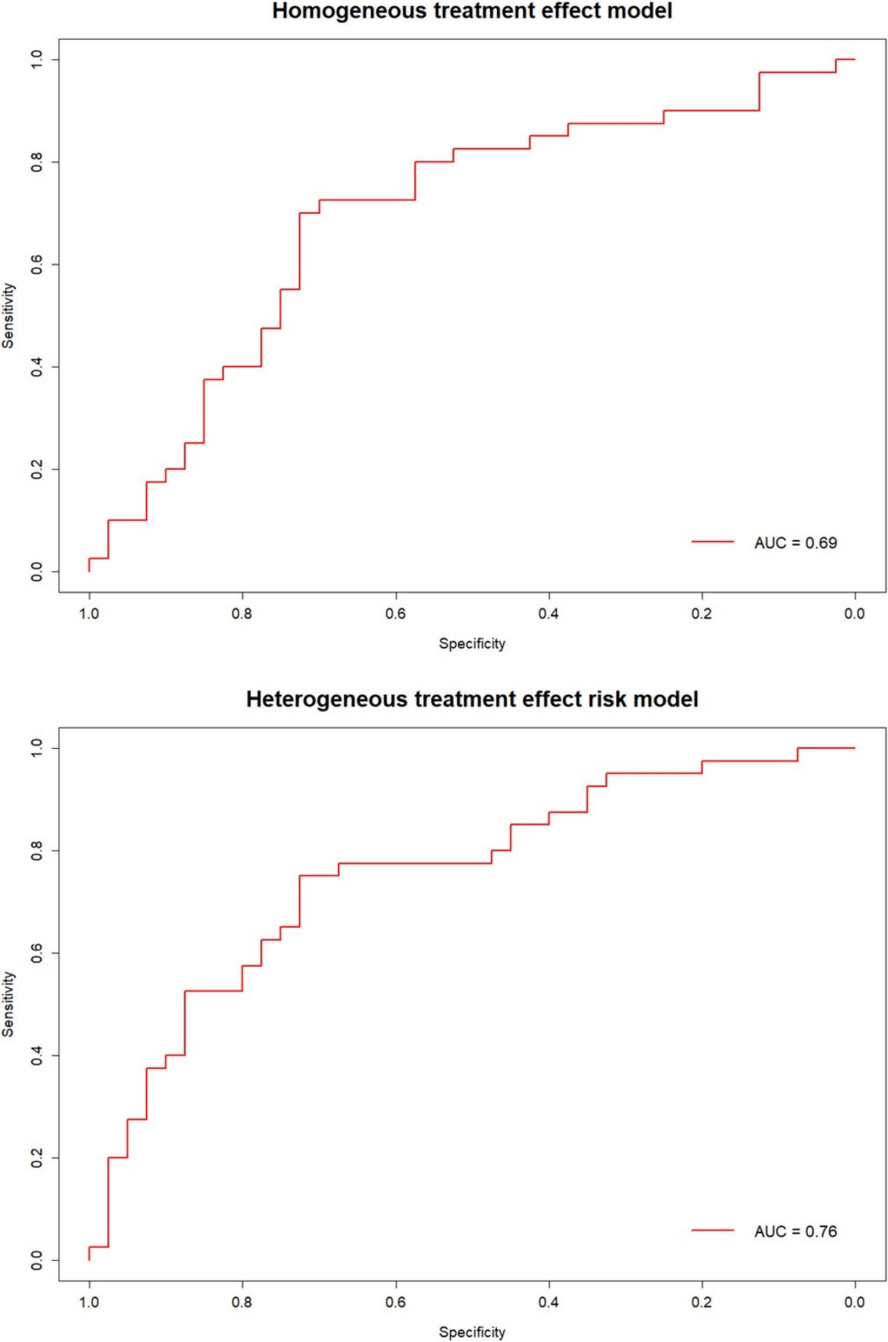
ROC curve of the prediction models. Legend: λ was estimated using nested cross-validation

The AUC-ROC for the homogeneous treatment effect model after cross-validation was 0.69 (Fig. 2). Using this modelling strategy, methotrexate was predicted to be most effective for all DN patients, and etanercept was predicted to be most effective for all DF patients. The differences in predicted probabilities of response were small: 0.5% to 0.6% in favour of methotrexate in DN patients and 6.5% to 7.8% in favour of etanercept in DF patients.

### Heterogeneous treatment effect: risk modelling

For the risk model approach, AUC-ROC after cross validation was 0.76 (Fig. 2). In DN patients, the predicted probability of response to tofacitinib compared to methotrexate ranged from 13.4% lower to 1.7% higher. Methotrexate treatment had the highest predicted probability of response in 85% of DN patients. For DF patients, the predicted probability for tofacitinib was 6.7% to 7.8% lower compared to etanercept, so etanercept had the highest probability of response in all DF patients.

### Heterogeneous treatment effect: effect modelling

No interactions between predictor variables and treatment were selected. As such, this approach led to the homogeneous treatment effect model described above.

### Comparison of prediction models

Based on the AUC-ROC and on the variation in differences in predicted probabilities of response for tofacitinib versus the compared treatment, the risk modelling approach was deemed the most suitable. The regression coefficients for the variables that are used in this model are provided in Table 2 and Table 3. The range of differences in probability of response for the compared treatments are provided in Table 4.

**Table 2:**
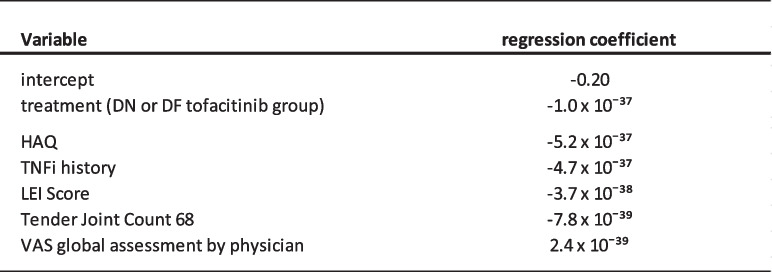
Variables used to calculate the linear predictor

The intercept and regression coëfficients of the general prediction model to predict response for tofacitinib patiënts (DN and DF patients) are provided here. The Linear Predictor (LP) variable of the risk model was calculated as a function of these regression coefficients multiplied by the values of the corresponding predictor variable for individual patients. The intercept and treatment group were not used to calculate the linear predictor. DN patient: disease modifying anti-rheumatic drug naïve patient; DF patient: patients with insufficient response to conventional synthetic disease modifying anti-rheumatic drugs; HAQ: Health Assessment Questionnaire; LEI: Leeds Enthesitis Index; VAS: Globas Analogue Scale; TNFi:

**Table 3:**
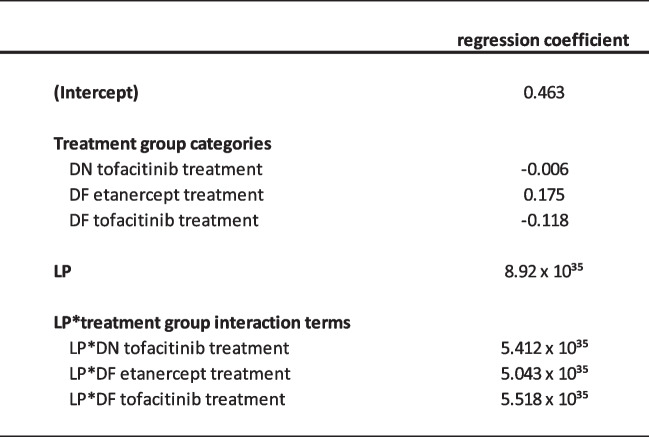
Variables of the risk model

The intercept and regression coëfficients that can be used to calculate the probability of achievement of MDA after 16 weeks are provided here. Methotrexate treatment in the DMARD naïve group was used as a reference category. DN: DMARD naïve; DF: DMARD failure; LP: linear predictor variable

**Table 4:**
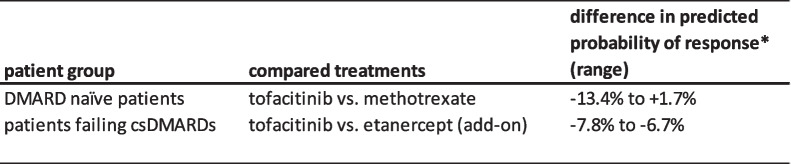
Predicted probability of response for the compared treatments

* calculated by the predicted probability of achievement of MDA if tofacitinib is assigned minus the predicted probability of achievement of MDA if methotrexate or etanercept is assigned, for each individual patient. A positive value is in favour of tofacitinib, a negative value is in favour of the standard of care

## Discussion

We aimed to develop a model to compare treatment response for tofacitinib versus methotrexate or etanercept for individual patients, using clinical data from the TOFA-PREDICT trial. Of the three prediction approaches we assessed, risk modelling showed the best results, with a reasonable AUC-ROC (0.76) and with clinically relevant differences in predicted response between the compared treatments, especially in DMARD naïve (DN) patients. Here we will discuss the potential clinical impact of the prediction model, and review the role of the predictive variables that were incorporated.

### Potential clinical impact of the prediction model

The final model (risk modelling approach) predicted higher predicted probability of response to methotrexate (compared to tofacitinib) in 85% of DN patients. The differences in predicted probability (tofacitinib versus methotrexate) were ranging from 13.4% lower to 1.7% higher. Such differences between predicted efficacy of compared treatments could support treatment decisions in clinical practice. However, the model should first be validated in an external cohort.

Our model predicts effectiveness of treatment, but the treating clinician should also take associated risks of treatment options into account. Considering the ORAL surveillance study, cardiovascular and malignancy risk factors should be taken into consideration when treatment is started. Post hoc analyses of PsA patients have identified specific subpopulations with a different relative risk for tofacitinib versus TNFi. [25] For malignancies (excluding non-melanoma skin cancer), a higher risk with tofacitinib was confined to patients ≥ 65 years of age and/or long-time current/past smokers. For major adverse cardiac events, it was confined to patients with a history of atherosclerotic cardiovascular disease. A careful risk–benefit comparison should be made for any treatment option, and we believe that our prediction model, when validated, could inform such an assessment for individual PsA patients. [28].

### Predictive variables for treatment response

An interesting finding is the selection of the HAQ score as a prominent predictor for MDA. It was selected as relevant predictor in the complete cohort, and the tofacitinib and etanercept subgroups. However, this questionnaire is not routinely used in outpatient settings, and it may correlate with other more easily and commonly acquired patient reported measures such as VAS pain or VAS global assessment. We therefore conducted an additional predictor selection step excluding the HAQ. In this analysis, less and no alternative predictors were selected, and the predictive performance of the model declined. Given HAQ’s strong predictive value in our initial analyses and its relevance as a measure of patient-reported disability, its inclusion in the model was deemed justified.

High HAQ score (indicating increased functional disability of the patient) has been shown in other PsA studies to be a predictor of response mostly for TNFi treatment. [11, 13, 15] We hypothesised that there could be several explanations for the association between high HAQ scores and non-achievement of MDA. High HAQ scores could partly be associated with structural joint damage or with nociplastic pain. These factors could result in not achieving MDA despite a low disease activity (i.e. inflammation). Response to DMARD treatment would therefore not be accurately represented by MDA score in these cases. Several studies report associations between HAQ, structural joint damage, fibromyalgia/nociplastic pain and MDA. [26–29] Another explanation could be a correlation of high HAQ scores with severe joint inflammation, in which case MDA might not (yet) be achieved within 16 weeks, even if the treatment is effective. However, in the development cohort there was no strong correlation between HAQ score and the swollen joint count 66 (Spearman rank correlation coefficient 0.176, *p* = 0.118). For the purposes of this study the week 16 MDA remains a suitable primary outcome in line with a treat to target strategy.

Compared to the HAQ score that was selected as a predictor for response to multiple treatments, the other variables in the model were selected for response to one specific treatment. Increased LEI score was selected as a predictor specifically for methotrexate non-response. Little is reported regarding prediction of treatment response in PsA and entheseal involvement. However, treatment recommendations for PsA with predominant enthesitis tend towards bDMARDs since there is a lack of strong evidence for effectiveness of methotrexate. [4, 7, 8] Additionally, increased Tender Joint Count (TJC) was a predictor for non-response to methotrexate. This could be partially explained because achievement of MDA within 16 weeks is more difficult in patients with a larger number of inflamed joints, with a higher TJC. However, the swollen joint count was not a selected predictor, suggesting that other non-inflammatory causes of joint tenderness could also explain non-response to DMARD treatment. Lower VAS global assessment by physician was another predictor for non-response in the methotrexate group. Possibly, lower VAS global assessment scores were given if the physician deemed overall inflammation to be mild, explaining insufficient response to methotrexate. Lastly, TNFi history was identified as a relevant predictor for etanercept response. This seems logical, since the effectiveness of a second TNFi is reported to be markedly decreased compared to response rates of the first TNFi. [30].

### Limitations of the study

An important limitation is the relatively small study population, specifically the number of patients per treatment arm (*n* = 20), which reduces the possibility of finding important predictors for response for that specific treatment. We reduced the risk of overfitting the data as much as possible by using penalised regression methods, and by using nested cross-validation (CV) for the estimation of λ for both elastic net and Ridge regression analyses. By choosing λ_1se_ as penalty, the models were more regularised. In addition to CV, our aim is to externally validate the clinical risk model in a later stage, when the validation cohort of the TOFA-PREDICT study is completed.

Another limitation is that randomised clinical trial data was used for the development of a prognostic prediction model. Selective patient inclusion and extraneous trial effects could limit the applicability of such a model in clinical practice. [31] To mitigate these factors, the eligibility criteria of the study were designed to represent a regular outpatient population as much as possible, with the primary goal of developing a prognostic prediction model that is clinically applicable.

### Future analysis plans for the TOFA-PREDICT study

The derived prediction model shows that clinical data alone allows for the prediction of treatment response to different treatments. However, before the proposed model can be used to aid treatment decisions, validation is required. We aim to do this using the TOFA-PREDICT validation cohort.

The performance of the clinical prediction model raises the question whether incorporating additional biomarkers could enhance the predictions. The TOFA-PREDICT study ultimately aims to create a prediction model integrating various omics platforms and imaging parameters to assess their predictive value beyond our existing clinical risk model. The study's ultimate objective is to develop a comprehensive multi-omics prediction model to aid personalised treatment in PsA.

## Supplementary Information

Supplementary Material 1. Figure S1. The TOFA-PREDICT study design. The TOFA-PREDICT study design. As previously published (open protocol). In the current analysis, the DMARD-naïve group is referred to as csDMARD naïve (DN) patients, and the DMARD-NR group is referred to as csDMARD failure patients. DMARD: disease modifying antirheumatic drug; CASPAR criteria: classification of psoriatic arthritis criteria; NR: non-responder; csDMARD: conventional synthetic DMARD.

Supplementary Material 2. Information S2. Additional information cross-validation [32, 33].

Supplementary Material 3. Information S3. Additional information heterogeneous treatment effect risk modelling.

Supplementary Material 4. Figure S4. Cross-validation plots. Cross-validation plots. Leave one out cross-validated lambda parameter is plotted against binomial deviance for each outer cross-validated fold. Ridge regression cross-validation plots are shown for the homogeneous treatment effect model and the heterogeneous treatment effect risk model.

Supplementary Material 5. Figure S5. ROC curve of the prediction models including nested cross-validation. ROC curve of the prediction models including nested cross-validation. An inner LOOCV loop was used to tune λ and an outer LOOCV loop was used to determine the model performance. The resulting nested LOOCV λ was used for the final model. The ROC curve using λ estimated in the inner loop (grey dotted line), outer loop (blue dotted line), and after nested LOOCV (red line) are shown. The AUC-ROC becomes more conservative from inner loop to outer loop to the final model. LOOCV: leave one out cross validation.

## Data Availability

The datasets used and/or analyzed during the current study are available from the corresponding author on reasonable request.

## Abbreviations

DMARDs: Disease-Modifying Antirheumatic Drugs
csDMARD: Conventional synthetic DMARD
PsA: Psoriatic Arthritis
GRAPPA: Group for Research and Assessment of Psoriasis and Psoriatic Arthritis
EULAR: European alliance of associations for rheumatology
ACR: American College of Rheumatology
TNFi: Tumour Necrosis Factor alpha inhibitors
HAQ: Health Assessment Questionnaire
CRP: C-reactive Protein
BMI: Body Mass Index
DAPSA: Disease Activity Index in Psoriatic Arthritis
bDMARD: Biological DMARD
JAKi: Janus-Kinase inhibitor
DN patients: CsDMARD naïve
DF patients: CsDMARD failure
BSA: Body Surface Area
LEI: Leeds Enthesitis Index
LP: Linear Predictor
AUC-ROC: Area Under the Curve of the Receiver Operating Characteristics
TJC: Tender Joint Count
tsDMARD: Targeted synthetic DMARD
CV: Cross-validation
VAS: Visual Analog Scale
LOOCV: Leave One Out Cross-validation
CASPAR criteria: ClASsification of Psoriatic Arthritis
