# Supplementary figures and images for: The development of a clinical prediction model for response to methotrexate, tofacitinib, and etanercept in patients with Psoriatic Arthritis
Source: Arthritis Res Ther. 2025 Oct 27;27:197. doi: 10.1186/s13075-025-03660-2 (PMC12560565; doi:10.1186/s13075-025-03660-2)

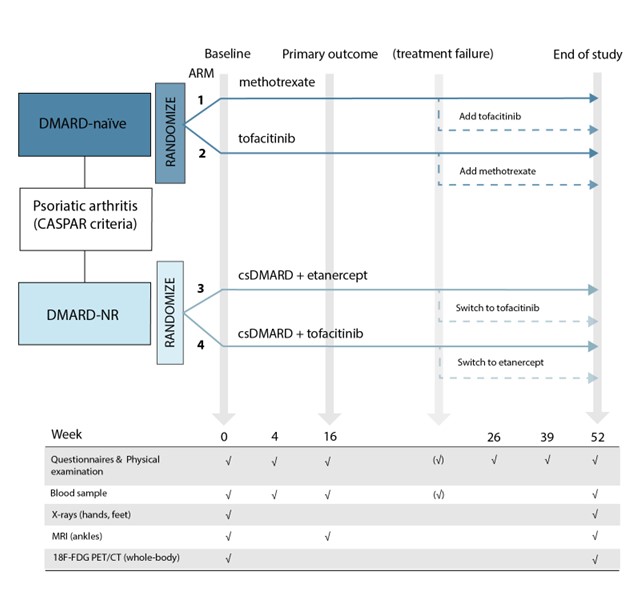

Supplement: Supplementary file 1 — Supplementary Material 1. Figure S1. The TOFA-PREDICT study design. The TOFA-PREDICT study design. As previously published (open protocol). In the current analysis, the DMARD-naïve group is referred to as csDMARD naïve (DN) patients, and the DMARD-NR group is referred to as csDMARD failure patients. DMARD: disease modifying antirheumatic drug; CASPAR criteria: classification of psoriatic arthritis criteria; NR: non-responder; csDMARD: conventional synthetic DMARD. [file 13075_2025_3660_MOESM1_ESM.jpg]

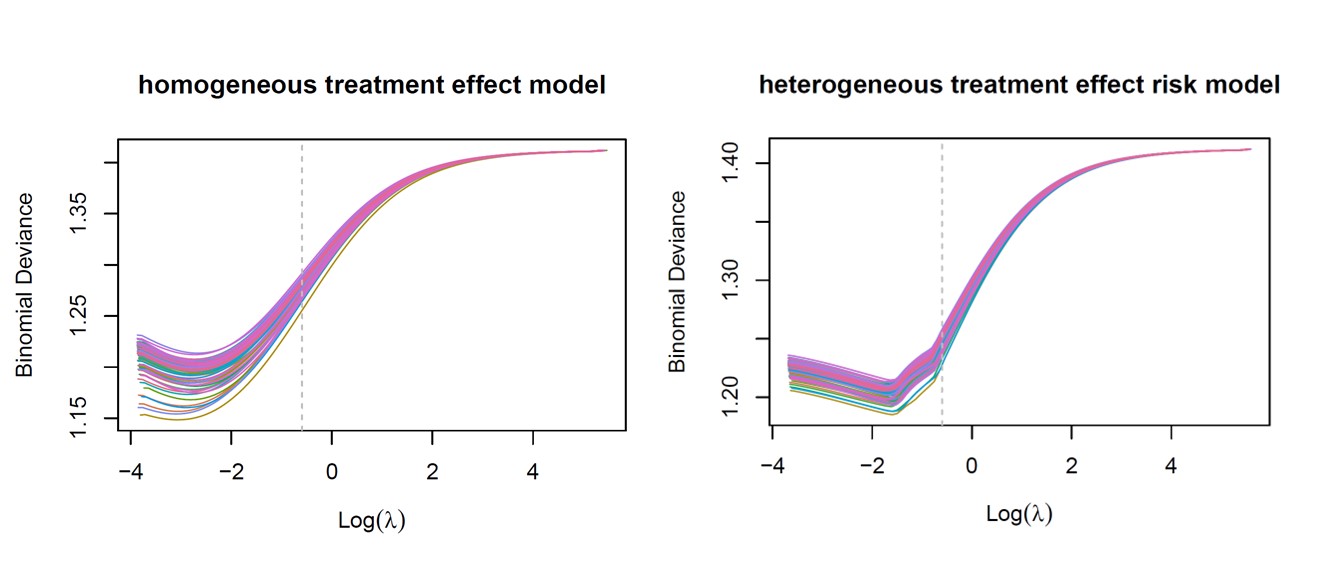

Supplement: Supplementary file 4 — Supplementary Material 4. Figure S4. Cross-validation plots. Cross-validation plots. Leave one out cross-validated lambda parameter is plotted against binomial deviance for each outer cross-validated fold. Ridge regression cross-validation plots are shown for the homogeneous treatment effect model and the heterogeneous treatment effect risk model. [file 13075_2025_3660_MOESM4_ESM.jpg]

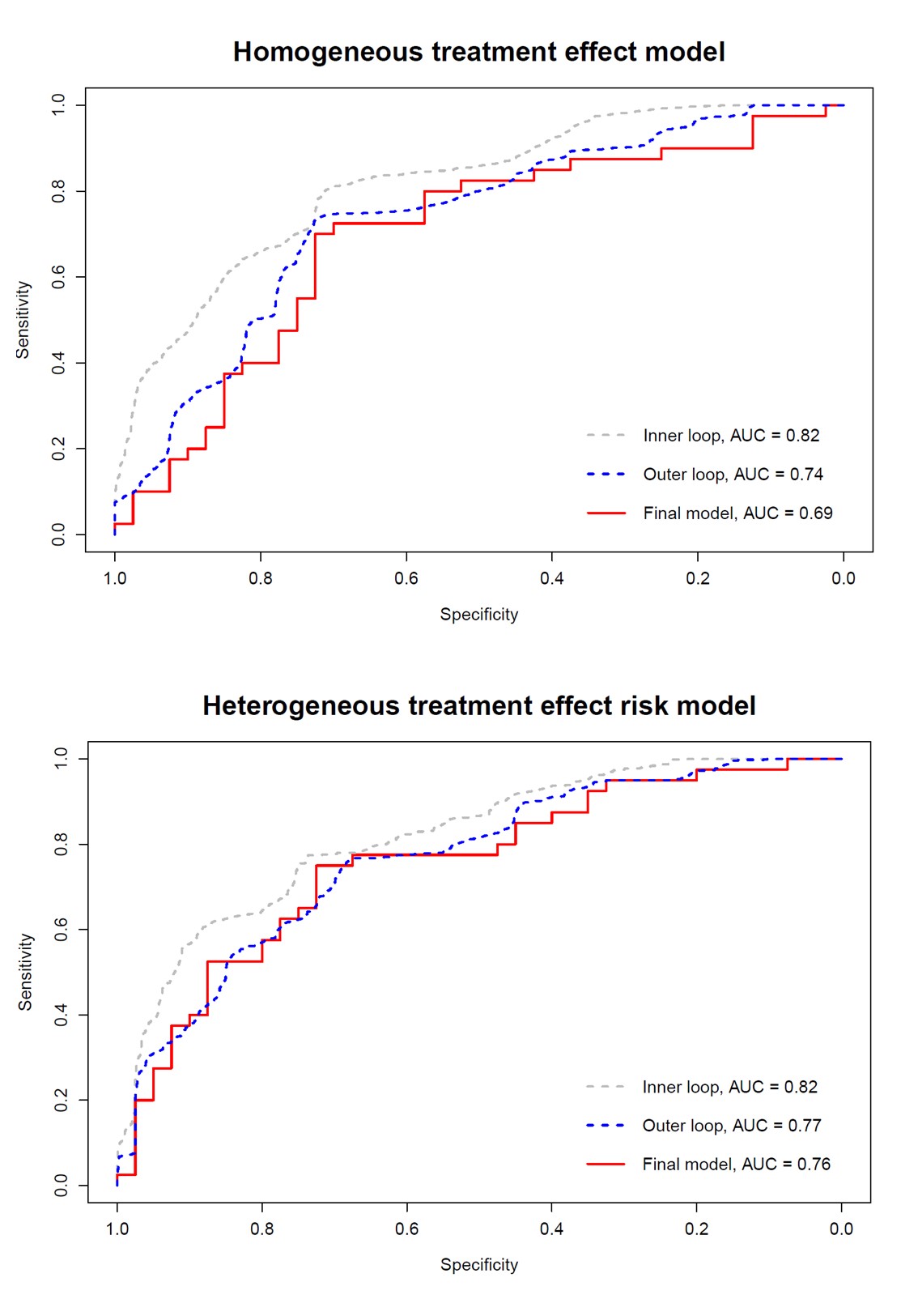

Supplement: Supplementary file 5 — Supplementary Material 5. Figure S5. ROC curve of the prediction models including nested cross-validation. ROC curve of the prediction models including nested cross-validation. An inner LOOCV loop was used to tune λ and an outer LOOCV loop was used to determine the model performance. The resulting nested LOOCV λ was used for the final model. The ROC curve using λ estimated in the inner loop (grey dotted line), outer loop (blue dotted line), and after nested LOOCV (red line) are shown. The AUC-ROC becomes more conservative from inner loop to outer loop to the final model. LOOCV: leave one out cross validation. [file 13075_2025_3660_MOESM5_ESM.jpg]
