# Supplementary material for: The development of a clinical prediction model for response to methotrexate, tofacitinib, and etanercept in patients with Psoriatic Arthritis
Source: Arthritis Res Ther. 2025 Oct 27;27:197. doi: 10.1186/s13075-025-03660-2 (PMC12560565; doi:10.1186/s13075-025-03660-2)
Supplement: Supplementary file 2 — Supplementary Material 2. Information S2. Additional information cross-validation [32, 33]. [file 13075_2025_3660_MOESM2_ESM.docx]

**Supplementary Information S2**. Additional information elastic net regression and cross-validation.

Elastic net regression and Ride regression are penalised/regularised regression analysis methods, using cross-validation (CV) to derive a penalty (λ). This penalty is used to shrink regression coefficients towards zero.

In elastic net regression, some regression coefficients will shrink completely to zero, leading to the exclusion of the variable from the model. This way, relevant predictor variables are selected. Ridge regression is a similar method to elastic net regression, but no coefficients are fully shrunken to zero. As a result, all selected variables are kept in the model.

Both elastic net and Ridge analyses were performed using the R package *glmnet* (version 4.1-6). (32) We used *nestedcv* (version 0.7.8), an R package for nested CV for regularised models, to estimate the optimal λ.(33) We used nested cross-validation with an inner loop to tune λ, and outer loop to determine model performance. For the predictor selection, we used elastic net analysis with 5-fold inner loop and 5-fold outer loop cross validation. The optimal alpha (a regularisation strength parameter) was selected from a range of 0.5 to 0.8. To increase stability of the selection in the relatively small treatment subgroups, the predictor selection was repeated 100 times, and only predictors that were selected at least 70 times were kept. For the Ridge analyses, we chose to use leave-one out cross-validation for both inner and outer loops. This way we aimed to limit overfitting of the model, and use a maximum number of patients to develop the model. We used λ_1se_ (the largest value for λ within one standard error of the λ with a minimum mean cross-validated error) for the selection of predictor variables.
