# Supplementary material for: The development of a clinical prediction model for response to methotrexate, tofacitinib, and etanercept in patients with Psoriatic Arthritis
Source: Arthritis Res Ther. 2025 Oct 27;27:197. doi: 10.1186/s13075-025-03660-2 (PMC12560565; doi:10.1186/s13075-025-03660-2)
Supplement: Supplementary file 3 — Supplementary Material 3. Information S3. Additional information heterogeneous treatment effect risk modelling. [file 13075_2025_3660_MOESM3_ESM.docx]

**Supplementary Information S3.** Additional information heterogeneous treatment effect risk modelling.

An ideal setting for risk modelling would be to compare a homogeneous intervention and a homogeneous control group. However, the TOFA-PREDICT study population contains two subgroups (DN patients and DF patients), where the “control group” is different between the two subgroups (methotrexate and etanercept), and the “intervention group” (tofacitinib) consists of both DN and DF patients. To emphasise the comparison between tofacitinib and standard of care, we estimated the LP in the intervention groups (DN and DF patients receiving tofacitinib) and to subsequently add treatment group (including all treatment arms, e.g. both intervention and control groups) and LP*treatment group interaction terms. In other words, the LP of the risk model was ‘trained’ by the intervention treatment, but prediction of response for all four treatment arms was enabled by adding treatment group as a predictor, as well as all the LP*treatment group interaction terms.
